# The Clinical Picture of Psychosis in Manifest Huntington's Disease: A Comprehensive Analysis of the Enroll-HD Database

**DOI:** 10.3389/fneur.2018.00930

**Published:** 2018-11-06

**Authors:** Natalia P. Rocha, Benson Mwangi, Carlos A. Gutierrez Candano, Cristina Sampaio, Erin Furr Stimming, Antonio L. Teixeira

**Affiliations:** ^1^Department of Psychiatry and Behavioral Sciences, McGovern Medical School, The University of Texas Health Science Center at Houston (UTHealth), Houston, TX, United States; ^2^HDSA Center of Excellence at University of Texas Health Science Center at Houston, Houston, TX, United States; ^3^The Nathan S. Kline Institute for Psychiatric Research, Orangeburg, NY, United States; ^4^CHDI Management, CHDI Foundation, Princeton, NJ, United States; ^5^Department of Neurology, McGovern Medical School, The University of Texas Health Science Center at Houston, Houston, TX, United States

**Keywords:** Huntington's disease, psychosis, behavior, Enroll-HD, machine learning

## Abstract

**Background:** Psychotic symptoms have been under-investigated in Huntington's disease (HD) and research is needed in order to elucidate the characteristics linked to the unique phenotype of HD patients presenting with psychosis.

**Objective:** To evaluate the frequency and factors associated with psychosis in HD.

**Methods:** Cross-sectional study including manifest individuals with HD from the Enroll-HD database. Both conventional statistical analysis (Stepwise Binary Logistic Regression) and five machine learning algorithms [Least Absolute Shrinkage and Selection Operator (LASSO); Elastic Net; Support Vector Machines (SVM); Random Forest; and class-weighted SVM] were used to describe factors associated with psychosis in manifest HD patients.

**Results:** Approximately 11% of patients with HD presented history of psychosis. Logistic regression analysis indicated that younger age at HD clinical diagnosis, lower number of CAG repeats, history of [alcohol use disorders, depression, violent/aggressive behavior and perseverative/obsessive behavior], lower total functional capacity score, and longer time to complete trail making test-B were associated with psychosis. All machine learning algorithms were significant (chi-square *p* < 0.05) and capable of distinguishing individual HD patients with history of psychosis from those without a history of psychosis with prediction accuracy around 71–73%. The most relevant variables were similar to those found in the conventional analyses.

**Conclusions:** Psychiatric and behavioral symptoms as well as poorer cognitive performance were related to psychosis in HD. In addition, psychosis was associated with lower number of CAG repeats and younger age at clinical diagnosis of HD, suggesting that these patients may represent a unique phenotype in the HD spectrum.

## Introduction

Huntington's disease (HD) is traditionally classified as a movement disorder as its formal diagnosis is based on the unequivocal presence of otherwise unexplained extrapyramidal motor symptoms, for example chorea, dystonia, bradykinesia and rigidity (1, 2). Chorea is the most prominent symptom in the early stages of adult- or late-onset HD. Incoordination, bradykinesia and rigidity tend to predominate in early-onset HD and in the late stages of the more common adult-onset HD (1). While the presence of motor symptoms is required for the clinical diagnosis of HD, cognitive impairment is also a core characteristic of the disease, and can emerge years before the diagnosis (3). Along with motor and cognitive changes, psychiatric issues complete the triad of signs and symptoms that characterize HD. Psychiatric symptoms can be present across all stages of HD, even preceding the onset of motor impairment. Although not universal, they are common and may be a cause of significant distress in HD. Psychiatric manifestations in HD include depression, irritability, apathy, obsessions, and occasionally psychosis (4).

Psychosis is defined by the presence of delusions and/or hallucinations (5). The prevalence of psychotic symptoms in HD patients is variable, ranging from 3 to 11% (6). Psychosis can be very distressful for both individuals with HD and their caregivers (7). Interestingly, the presence of psychotic symptoms has defined a specific phenotype in some Huntington pedigrees. In these cases, psychosis was the most prominent symptom and predated motor and cognitive changes in most affected member across generations (8).

Psychotic symptoms have been under-investigated in HD and research is needed in order to elucidate the characteristics potentially linked to the unique phenotype of HD patients presenting with psychosis. Therefore, the current study was carried out to evaluate the factors associated with psychosis in a large database of people with HD. Taking advantage of the Enroll-HD database (9), we used both conventional statistical analyses and multivariate machine learning methods to describe the factors associated with psychosis in HD. The results of this study might contribute to the understanding of psychosis in HD and, ultimately, improving the management of these patients.

## Methods

### Study design and participants

This study was based on information provided by the Enroll-HD database (9). Enroll-HD is a worldwide longitudinal observational study whose sites are located in North America, Latin America, Europe, Australia and New Zealand. Among other goals, Enroll-HD was designed to provide information about the dynamic phenotypic spectrum of HD and to promote the acquisition of knowledge about standards of care to inform clinical decisions, improving the health outcomes for the participant/family unit (9).

**Figure 1:**
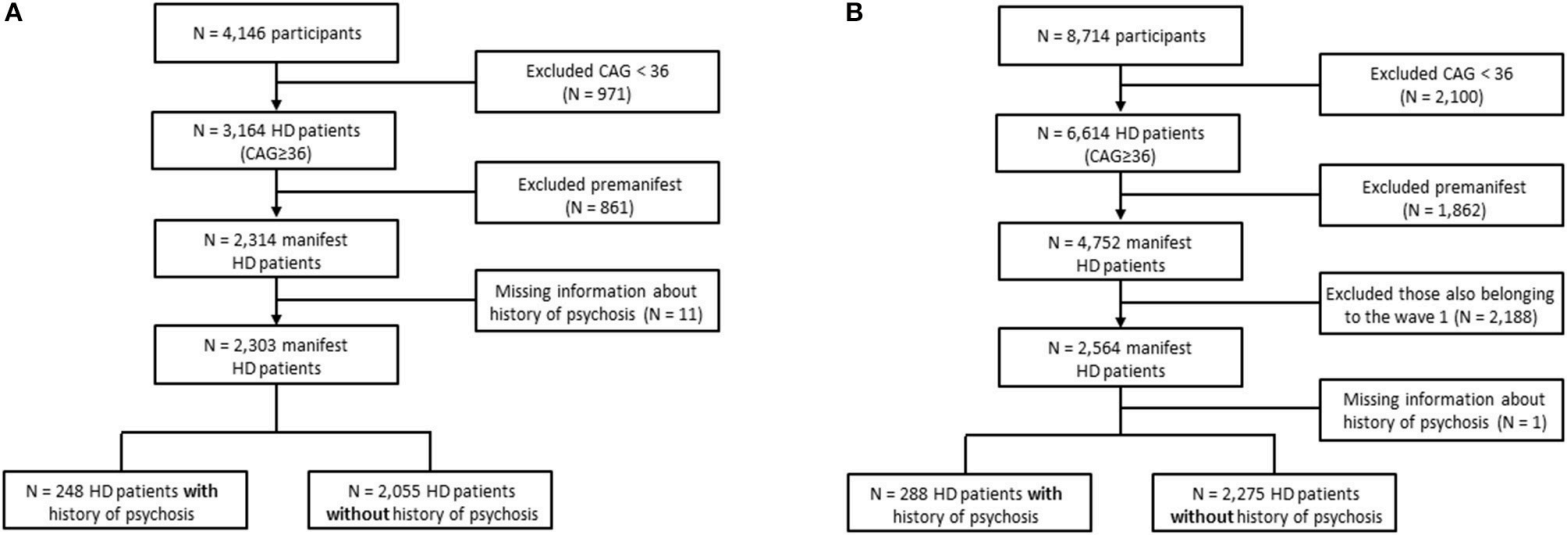
Flowchart showing participants' selection. **(A)** Conventional statistics and machine learning algorithms were applied to evaluate predictors of psychosis in the periodic dataset containing Enroll-HD participants which meet the criteria for inclusion into the dataset as of November 1, 2015 (Wave 1 sample). **(B)** Wave 2 dataset composed of new Enroll-HD participants whose information was released by the Enroll-HD as of October 31, 2016 (PDS3) used for validating the machine learning algorithms.

We used the periodic dataset containing Enroll-HD participants which meet the criteria for inclusion into the dataset as of November 1, 2015 (PDS2, Wave 1 sample). Cross-sectional data from baseline visits was obtained, with the data set being composed of 4,146 participants. From these, we excluded 971 participants whose number of CAG repeats was <36 (455 genotype negative and 516 family controls). The participants with a genetic diagnosis of HD (i.e., a CAG repeat length on the larger allele >=36) were divided into premanifest and manifest subjects. According to the Enroll-HD data handling manual, participants were classified as premanifest if they had the gene expansion for HD (larger CAG allele ≥36) but no clinical diagnosis of HD, [i.e., diagnostic confidence level (DCL) <4 on question 17 of the Unified Huntington's Disease Rating Scale (UHDRS)]. The manifest group was composed by participants with the gene expansion for HD (larger CAG allele ≥36) *and* a clinical diagnosis of HD based on the presence of unequivocal motor signs, i.e., DCL from the UHDRS = 4. A comparison between premanifest (*N* = 861) and manifest (*N* = 2,314) subjects revealed that while only 1.3% of premanifest patients presented history of psychosis, this number was much higher when considering manifest subjects (10.8%, *p* < 0.001, Fisher's exact test). Hence, we decided to run the analyses considering only manifest subjects. Figure 1A summarizes the exclusion process we applied in this study.

### Searching for factors associated with psychosis: conventional statistical analysis

First, we performed univariate analyses in order to investigate differences between HD patients with (*N* = 248) and without (*N* = 2,055) history of psychosis. Associations between dichotomous variables were assessed with the Fisher's exact test. All continuous variables were tested to assess whether they follow a Gaussian distribution using the Shapiro-Wilk normality test. Two groups (history of psychosis vs. no history of psychosis) were compared using the Mann–Whitney *U*-test since data were determined to not follow a normal distribution. Then, a binary logistic regression was performed to determine which variables (among general clinical characteristics, medical history of substance abuse and psychiatric and behavioral problems, motor and functional capacity and cognitive performance) were significant associated with history of psychosis. A backward stepwise regression was used and all variables described in Table 1 were included in the initial model, except those exhibiting some degree of interdependency: (i) mother OR father affected by HD; and (ii) variables representing history of abuse of specific drugs (marijuana, heroin, cocaine, etc.), since they are dependent on the variable “history of drugs abuse.” Therefore, the following variables were included in the initial model: age, sex, age at motor symptoms onset, age at HD clinical diagnosis (based on the presence of unequivocal motor signs, DCL = 4), whether the mother was affected, number of CAG repeats, medical history of (alcohol use disorders, smoking, drugs abuse, depression, irritability, violent/aggressive behavior, apathy, perseverative/obsessive behavior, cognitive impairment, suicidal ideation), total motor score (TMS), total functional capacity (TFC) score, symbol digit modalities test (SDMT) (number of correct responses), verbal fluency test (number of correct responses in 1 min), Stroop interference test (number of correct responses), trail making test (TMT) parts A and B (time to complete and number of correct responses) and mini-mental state examination (MMSE) score. The backward stepwise selection was automatically performed using the SPSS software version 25.0 (SPSS Inc., Chicago, IL, USA) and the removal testing was based on the probability of the likelihood-ratio statistic based on conditional parameter estimates. The goodness of fit of the logistic regression model was assessed by the Hosmer-Lemeshow test as well as a Receiver Operating Characteristic (ROC) curve.

**Table 1:** Demographics and clinical characteristics of manifest patients with Huntington's disease (HD) with and without history of psychosis.

	**Psychosis history**		***P*-value**
	**No (*N* = 2,055)**	**Yes (248)**	
Age in years [mean ± SD (median)]	52.4 ± 11.7 (53)	53.6 ± 12.6 (53.5)	0.18^b^
Sex (% female)	50.2	52.0	0.32^a^
Age at motor symptoms onset [mean ± SD (median)]	45.6 ± 11.6 (46)	44.1 ± 12.0 (45)	0.15^b^
Age of clinical HD diagnosis [mean ± SD (median)]	48.3 ± 12.1 (48)	47.01 ± 12.5 (47)	0.21^b^
Mother affected (%)	47.3	41.8	0.06^a^
Father affected (%)	46.7	52.3	0.06^a^
CAG repeats [mean ± SD (median)]	44.0 ± 3.7 (43)	43.9 ± 3.9 (43)	0.47^b^
Medical history of:			
Alcohol use disorders	9.3	17.3	<0.0001^a^
Smoking	49.0	51.8	0.22^a^
Drugs abuse	9.7	13.7	0.04^a^
Marijuana	86.0	88.2	0.49^a^
Heroin	5.5	11.8	0.16^a^
Cocaine	29.0	41.2	0.11^a^
Club drugs (ecstasy, GHB, roofies)	19.0	29.4	0.13^a^
Amphetamines	17.5	26.5	0.16^a^
Ritalin	1.5	0	0.62^a^
Hallucinogens	19.0	17.6	0.53^a^
Inhalants	1.0	5.9	0.10^a^
Opium	2.0	0	0.53^a^
Painkillers	7.0	5.9	0.58^a^
Barbiturates/sedatives	2.5	5.9	0.27^a^
Tranquilizers	1.5	2.9	0.47^a^
Depression (%)	63.8	87.0	<0.0001^a^
Irritability (%)	60.5	83.1	<0.0001^a^
Violent/aggressive behavior (%)	27.2	59.3	<0.0001^a^
Perseverative/obsessive behavior (%)	39.4	73.8	<0.0001^a^
Apathy (%)	52.4	76.2	<0.0001^a^
Cognitive impairment (%)	57.9	77.7	<0.0001^a^
Previous suicidal ideation (%)	23.2	42.5	<0.0001^a^
Total motor score [mean ± SD (median)]	38.1 ± 20.9 (35)	50.1 ± 25.0 (47)	<0.0001^b^
Total functional capacity [mean ± SD (median)]	8.2 ± 3.5 (9)	5.3 ± 3.5 (5)	<0.0001^b^
SDMT (total correct) [mean ± SD (median)]	23.5 ± 13.0 (22)	16.6 ± 13.5 (15)	<0.0001^b^
Verbal fluency test (category) – number of correct responses in1 min [mean ± SD (median)]	12.1 ± 5.7 (12)	9.4 ± 6.1 (9)	<0.0001^b^
Stroop Interference Test – number of correct responses [mean ± SD (median)]	24.2 ± 11.7 (24)	18.6 ± 12.6 (17)	<0.0001^b^
TMT-A time to complete [mean ± SD (median)]	71.8 ± 52.9 (55)	104.1 ± 70.4 (83)	<0.0001^b^
TMT-A number of correct responses [mean ± SD (median)]	24.1 ± 4.4 (25)	22.4 ± 7.1 (25)	<0.0001^b^
TMT-B time to complete [mean ± SD (median)]	151.2 ± 71.8 (141)	187.4 ± 66.0 (239)	<0.0001^b^
TMT-B number of correct responses [mean ± SD (median)]	21.2 ± 9.5 (25)	17.1 ± 9.6 (24)	<0.0001^b^
MMSE [mean ± SD (median)]	25.3 ± 4.1 (26)	22.2 ± 6.6 (24)	<0.0001^b^

*Only CAG ≥ 36 and manifest HD subjects (N = 2,303) from Enroll-HD which meet the criteria for inclusion into the dataset as of November 1, 2015*.

a *Fisher's exact test*;

b *Mann-Whitney test*.

*SD, Standard deviation; SDMT, Symbol Digit Modalities Test; TMT, Trail Making Test; MMSE, Mini–Mental State Examination*.

In order to validate our findings, the same variables included in the logistic regression model described above were tested in an independent cohort of patients with HD (Wave 2). Specifically, the Wave 2 data were composed of new Enroll-HD participants (*N* = 4,752 manifest subjects) whose information was released by the Enroll-HD as of October 31, 2016 (PDS3, Figure 1B).

Lastly, we evaluated the percentage of patients that presented pre-morbid psychosis (i.e., psychosis antedating the clinical diagnosis of HD) and the current presence of psychotic symptoms [defined as a score ≥1 in the psychosis subscale of the Problem Behaviors Assessment–short version (PBA-s)]. The PBA-s psychosis subscale is calculated as the sum of delusions / paranoid thinking score + hallucinations score (questions 9 and 10 of the PBA-s, respectively).

### Searching for variables capable of distinguishing individuals with and without psychosis: an individualized approach using multivariate machine learning

Machine learning—also known as pattern recognition—is a branch of computer science that involves developing algorithms that can learn from patterns of data, and subsequently able to make predictions on previously “unseen” observations (10). These algorithms are able to identify patterns of interactions among multiple variables and facilitate predictions and stratification of individual subjects' clinical outcomes (11). Machine learning has recently gained traction in biomedical studies due to their ability to analyze data from multiple observations and varied sources—also known as “big data” (11, 12). Machine learning approaches can result in highly accurate predictive models that support important clinical decisions such as selection of treatment options, preventive strategies, and prognosis orientations.

Machine learning algorithms are typically implemented in three stages: (1) algorithm training, (2) algorithm testing; and (3) validation. First, the data are divided into two groups (i.e., training and testing sets). Stage 1: the “training” set is used to train the algorithm and identify a set of optimal algorithm parameters. Stage 2: the “testing” set is used to examine whether the algorithm is able to generalize from the training set and calculate algorithm's prediction performance using accuracy, sensitivity and specificity metrics. Notably, it is a common practice to use a *k-*fold (i.e., *k* = 5 or 10) cross-validation method to separate algorithm training and testing data sets. Stage 3: once the algorithm has gone through the “training” and “testing” phase, it is evaluated using a “novel” evaluation data set—which was not included in the algorithm training or testing stages (13). In this study, we examined five machine learning algorithms: (i) Least Absolute Shrinkage and Selection Operator (LASSO) (14); (ii) Elastic Net (15); (iii) Support Vector Machines (SVM) (16); (iv) Random Forest (17); and (v) class-weighted SVM (18).

The same variables included in the Binary Logistic Regression (described in section Searching for Factors Associated With Psychosis: Conventional Statistical Analysis) were included in the machine learning approach. The main motivation here was to examine which demographic and clinical characteristics can *individually* distinguish between HD patients with and without psychosis. The machine learning algorithms were implemented using the Python programming language (19) through the Scikit-learn machine learning package (20). Missing data were imputed by replacing missing predictor variables with the mean. Predictor variables (Table 1) were normalized between zero and unity and together with corresponding categorical labels (0 – no history of psychosis; 1 – history of psychosis) used as an “input-target” pair for machine learning analyses.

Machine learning algorithms used in this study can be divided into three broad categories: (1) penalized linear regression (LASSO and Elastic Net), (2) Kernel-based (SVMs) and (3) Ensemble decision trees (random forests). Penalized linear regression methods use a classical linear regression approach albeit with additional penalty parameters to facilitate selection of most relevant variables or remove those that are redundant (21, 22). The penalized linear regression algorithms employed in this study use a logistic function to model probability and categorical outcomes (14, 15). On the other hand, Kernel-based methods use linear and non-linear kernel functions (e.g., polynomial, linear, and Gaussian) to “map” training data onto a higher dimensional space where a decision function able to separate both clinical groups is derived. Lastly, ensemble decision tree methods begin by constructing multiple decision trees which are subsequently combined by taking a majority (i.e., mode) of the predicted scores (17, 23).

#### The class imbalance problem

Class imbalance is a common problem in machine learning classification studies which happens when the number of observations in one class (e.g., no psychosis, *N* = 2,055) greatly exceeds the remaining class (e.g., psychosis, *N* = 238) (10, 23, 24). The class imbalance problem results in the machine learning algorithm being unable to generalize to previously unseen observations by largely assigning them to the majority class (24). Although there are multiple solutions that can mitigate the class imbalance problem (25), we used the majority class random under-sampling method (24, 25) and in the case of SVMs, a weighted SVM algorithm was also implemented.

**Figure 2:**
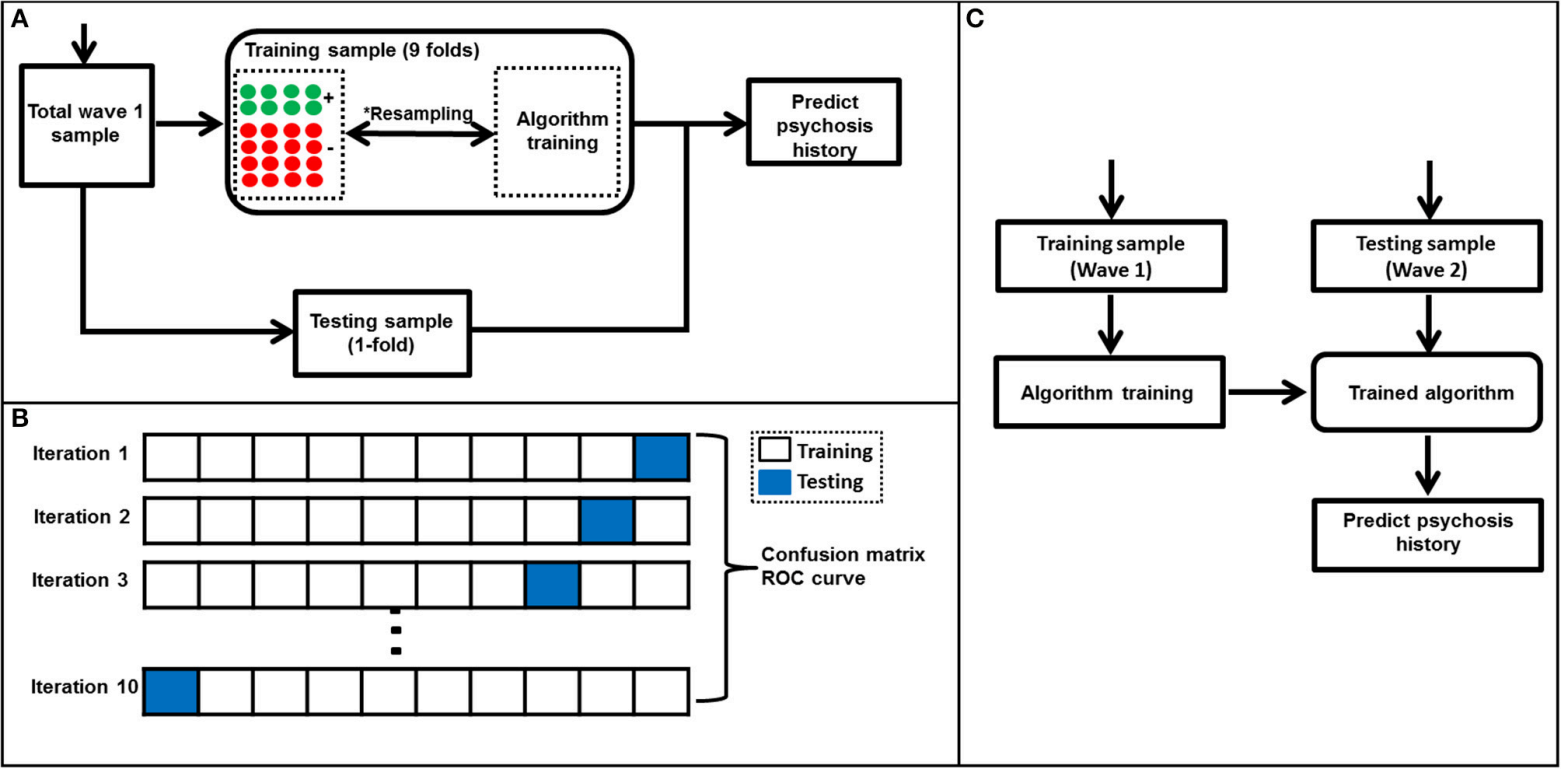
Algorithm training and testing process. **(A)** A flow diagram showing algorithm training and testing process. This process which included a *majority class undersampling step to mitigate the class imbalance problem was used in all algorithms except weighted SVM. The majority class undersampling process was repeated 5,000 iterations and predicted probabilities averaged over all iterations. Notably, the weighted SVM did not require a resampling step as it's able to mitigate for class imbalance by weighting the algorithm penalty parameter by the ratio of observations in each class. A standard 10-fold cross validation was used to separate training and testing samples in wave 1. **(B)** A representation of the 10-fold cross-validation process used in this study. First, the wave 1 sample was randomly separated into ten folds with nearly equal number of subjects in each fold. At every iteration (i.e., 1–10), a machine learning algorithm was trained using the training set and tested using the testing set (in blue). This process was repeated until all folds were left out of the training stage at-least once. Lastly, results were aggregated and used to generate a confusion matrix and ROC curve. The machine learning algorithms' ability to predict history of psychosis was examined using standard statistical metrics such as accuracy, specificity, sensitivity and area under ROC curve. **(C)** A flow diagram representing the machine learning algorithm validation using Wave 2 data. The algorithm was trained to predict individual subjects' history of psychosis using wave 1 data only and evaluated using wave 2 data.

Briefly, the majority class under-sampling method involves “under-sampling” the majority class (i.e., individuals without psychosis) which is followed by training a machine learning algorithm using a balance sample. In this study, this process was repeated 5,000 iterations and predictions aggregated as shown in Figure 2A. On the other hand, a weighted SVM algorithm mitigates the class imbalance problem by weighting the SVM penalty parameter with the corresponding ratio of observations in each class. For example, in this study the SVM penalty—also known as regularization parameter—was weighted using a ratio of psychosis vs. non-psychosis (i.e., 1:8). We used the weighted SVM algorithm as implemented in the Scikit-learn package (20) and explored in details elsewhere (26). Noticeably, the weighted SVM method did not require under-sampling of the majority class and, therefore, used all data during training which is a major strength as compared to the majority class random under-sampling method mentioned above (26).

#### Machine learning algorithm training, testing and validation

The training and testing of all machine learning algorithms were performed using a 10-fold cross-validation approach (27) which entailed subdividing the Wave 1 sample into 10 subsets. Therefore, in each iteration 9 subsets were used for training the algorithm while the remaining subset was used for testing. This process was repeated until all subsets were used for testing the at-least once (Figure 2B). Notably, all algorithm parameters were selected using a nested 10-fold cross-validation which excluded the test sample to avoid circularity or *double-dipping* (28, 29). The algorithms' ability to identify novel or previously “unseen” subjects as belonging to either psychosis or non-psychosis was quantified using prediction accuracy, sensitivity, specificity, positive predictive value (PPV) and negative predictive value (NPV) values. ROCs and the corresponding AUCs were computed. Chi-square statistical tests between actual and machine learning predicted labels were also calculated and considered significant when *p* < 0.05. Permutation-based *p*-values were calculated using the Scikit-learn package (20, 30) and significance set at *p* < 0.05.

Lastly, at the validation stage, the machine learning algorithms were trained using Wave 1 data and validated using a unique or “novel” Wave 2 dataset (Figure 2C). The validation step entailed training the algorithms using the Wave 1 sample by selecting parameters using a 10-fold cross-validation. Subsequently, the algorithms were tested using the Wave 2 sample. As above, prediction accuracy, specificity, sensitivity, PPV, NPV, ROC curve, and AUC were also computed.

## Results

### Conventional statistics results

Nearly 11% of motor manifest individuals with HD presented with a history of psychosis (*N* = 248 out of the 2,303). Demographics and clinical characteristics of manifest patients with HD with and without history of psychosis are shown in Table 1. The CAG trinucleotide repeat length was similar in both groups. Patients with a history of psychosis exhibited a higher frequency of behavioral problems and worse motor, functional capacity and cognitive scores than patients without a history of psychosis (Table 1).

Regarding the multivariate analysis, younger age at HD clinical diagnosis, lower number of CAG repeats, clinical history of [alcohol use disorders, depression, violent/aggressive behavior and perseverative/obsessive behavior], lower TFC score, and longer time to complete TMT-B (meaning worse cognitive performance) remained as significant factors associated with the history of psychosis in the final model (step 19). The results are presented in Table 2. The logistic regression model was significant [Hosmer-Lemeshow goodness of fit test (step 19): Chi-square = 11.4; *p* = 0.2] and the predicted variability resulted in an area under the curve (AUC) of 0.793 in the ROC analysis (Supplementary Figure 1A). The logistic regression results were corroborated by an external validation, since the relevant factors associated with psychosis in HD described above were very similar in the Wave 2 sample analysis [Supplementary Table 1; Hosmer-Lemeshow goodness of fit test (step 18): Chi-square = 7.2; *p* = 0.5. AUC = 0.816 in the ROC analysis (Supplementary Figure 1B)].

**Table 2:** Final logistic regression model (step 19) to define factors associated with psychosis in Huntington's disease (HD).

**Variable**							**95% CI for odds ratio**	
	**B**	**SE**	**Wald**	**df**	***p*-value**	**Odds ratio**	**Lower**	**Upper**
Age at clinical diagnosis	−0.51	0.016	9.749	1	0.002	0.951	0.921	0.981
Number of CAG repeats	−0.149	0.056	7.051	1	0.008	0.862	0.772	0.962
History of alcohol use disorders	0.568	0.323	3.084	1	0.079	1.764	0.936	3.324
History of depression	1.235	0.372	11.053	1	0.001	3.440	1.660	7.126
History of violent/aggressive behavior	0.711	0.246	8.358	1	0.004	2.036	1.257	3.297
History of perseverative/obsessive behavior	1.374	0.276	24.866	1	0.000	3.952	2.303	6.674
TFC score	−0.074	0.043	3.025	1	0.082	0.929	0.854	1.009
TMT-B (time to complete)	0.006	0.002	7.207	1	0.007	1.006	1.001	1.010

*The analysis considered only CAG ≥ 36 and manifest HD subjects from Enroll-HD which meet the criteria for inclusion into the dataset as of November 1, 2015 (Wave 1 sample; N = 2,303)*.

*CI, confidence interval; df, degrees of freedom; TFC, total functional capacity; TMT, trail making test; SE, standard error*.

Our additional analyses revealed that among HD patients with history of psychosis, 31.6% currently have psychotic symptoms and 55.3% were pre-morbid (i.e., the age of psychosis symptoms preceded the age of clinical diagnosis of HD). Among these patients, the psychotic symptoms started in mean 4.11 (±6.26) years before the clinical diagnosis of HD. In addition, we observed that patients with HD who had a history of psychosis had higher scores in all the behavioral subscales of the PBA-s in comparison with patients with no history of psychosis (Supplementary Table 2).

### Machine learning results

**Figure 3:**
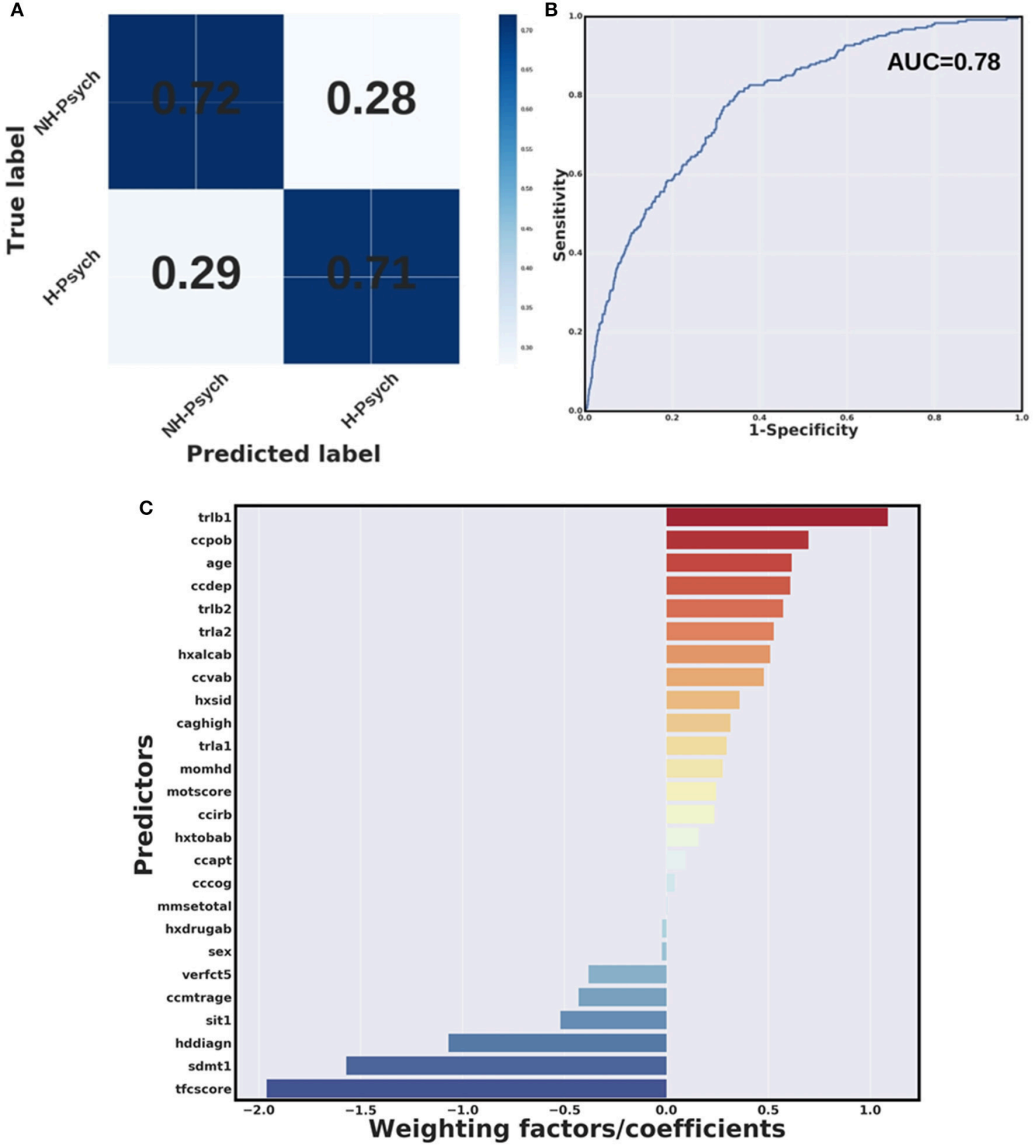
Weighted SVM algorithm. **(A)** Confusion matrix and **(B)** Receiver Operating Characteristic (ROC) curve for the weighted SVM algorithm in wave 1 data. H-Psych, history of psychosis; NH-Psych, no history of psychosis; AUC, area under the curve. **(C)** Bar graph containing coefficients or weighting factors assigned to each variable by the weighted SVM algorithm. tfcscore, total functional capacity score; trlb1, Trail making test (TMT)-B, time to complete; ccpob, history of perseverative/obsessive behavior; ccdep, history of depression; trlb2, TMT-B, total correct; trla2, TMT-A, total correct; hxalcab, history of alcohol use disorders; ccvab, history of violent/aggressive behavior; hxsid, previous suicidal ideation; caghigh, number of CAG repeats; trla1, TMT-A, time to complete; momhd, mother affected; motscore, total motor score; ccirb, history of irritability; hxtobab, history of smoking; ccapt, history of apathy; cccog, history of cognitive impairment; mmsetotal, mini-mental state examination, total score; hxdrugab, history of drugs abuse (hxdrugab); verfct5, verbal fluency test (animals), total correct in 1 min; ccmtrage, age at motor symptoms onset; sit1, Stroop interference test, total correct; hddiagn, age of clinical HD diagnosis; sdmt1, symbol digit modalities test, total correct.

All algorithms were capable of distinguishing individual HD patients with history of psychosis from those without a history of psychosis with prediction accuracy ranging from 71 to 73%. These results were established using a 10-fold cross-validation using the Wave 1 dataset only. All models were significant (chi-square *p* < 0.05, Table 3). A confusion matrix—which represents predicted labels against true labels (0 – no history of psychosis, 1 – history of psychosis) and a ROC curve for the weighted SVM algorithm are shown in Figures 3A,B, respectively.

**Table 3:** Algorithm performance in distinguishing between individuals who presented and who did not present history of psychosis (Wave 1 analysis, i.e., Enroll-HD participants which meet the criteria for inclusion into the dataset as of November 1, 2015).

**Algorithm**	**Balanced accuracy (specificity + sensitivity)/2**	**Classical accuracy**	**AUC**	**Specificity**	**Sensitivity**	**PPV**	**NPV**	**Chi-square/permutations *p*-value**
LASSO	0.72	0.70	0.79	0.69	0.75	0.71	0.73	*P* < 0.05
Elastic net	0.70	0.68	0.78	0.67	0.73	0.69	0.71	*P* < 0.05
SVM	0.72	0.70	0.72	0.70	0.74	0.71	0.73	*P* < 0.05
Random forest	0.71	0.71	0.77	0.71	0.72	0.71	0.71	*P* < 0.05
Weighted SVM	0.71	0.72	0.78	0.72	0.71	0.71	0.71	*p* < 0.05, *p* = 0.0002

*AUC, Area under the curve; LASSO, Least Absolute Shrinkage and Selection Operator; NPV, negative predictive value; PPV, positive predictive value; SVM, Support Vector Machines. Sensitivity and specificity represented correctly predicted history of psychosis (true positive rate) and correctly predicted no history of psychosis (true negative rate), respectively. The “classical” prediction accuracy was provided by taking the sum of true positives and true negatives divided by the total sample. Due to the class imbalance problem, a “balanced” accuracy was also calculated as the average of predicted sensitivity and specificity. PPV was calculated as the proportion of individuals predicted as having history of psychosis and who actually presented history of psychosis. NPV was calculated as the proportion of individuals who were predicted as not having history of psychosis and who actually did not present history of psychosis. Permutation-based p-values were calculated for the weighted SVM using the permutations tests method presented by Ojala et al. (30) and implemented in Scikit-learn*.

Figure 3C shows a bar graph representing weighting factors assigned to each variable by a weighted SVM based on their relevance in distinguishing individuals with and without history of psychosis. The most relevant variables in identifying individuals with and without history of psychosis were the TFC score, worse performance at cognitive tests (longer time to complete the TMT-B and lower scores at SDMT), as well as history of perseverative/obsessive behavior. In addition, younger age at clinical HD diagnosis was found to be relevant in distinguishing individuals with and without history of psychosis.

To further validate our results, the machine learning algorithms developed using the Wave 1 sample were tested on “novel” or previously unseen observations from the Wave 2 validation sample. Results obtained by the machine learning models in identifying subjects with and without history of psychosis from Wave 2 sample are shown in Table 4. All validation models were significant (chi-square *p* < 0.05, Table 4). In particular, the weighted SVM reported the best accuracy on the Wave 2 validation sample.

**Table 4:** Algorithm performance in distinguishing between individuals who presented and who did not present history of psychosis in Wave 2 data (new data released at the Enroll-HD periodic dataset containing information as of October 31, 2016).

**Algorithm**	**Balanced accuracy (specificity + sensitivity)/2**	**Classical accuracy**	**AUC**	**Specificity**	**Sensitivity**	**PPV**	**NPV**	**Chi-square/permutation test *p*-value**
LASSO	0.71	0.68	0.79	0.67	0.75	0.69	0.73	*P* < 0.05
Elastic net	0.70	0.67	0.77	0.66	0.74	0.69	0.72	*P* < 0.05
SVM	0.72	0.69	0.79	0.68	0.75	0.70	0.73	*P* < 0.05
Random forest	0.71	0.68	0.78	0.69	0.73	0.7	0.72	*P* < 0.05
Weighted SVM	0.72	0.70	0.79	0.69	0.75	0.71	0.73	*p* < 0.05, *p* = 0.0004

*AUC, Area under the curve; LASSO, Least Absolute Shrinkage and Selection Operator; NPV, negative predictive value; PPV, positive predictive value; SVM, Support Vector Machines*.

## Discussion

To our knowledge, this is the most comprehensive study evaluating factors associated with psychosis in HD. Using conventional statistical analyses, a history of depression, violent/aggressive behavior, perseverative/obsessive behavior and excessive alcohol use were, among the behavioral related variables, the significant variables associated with psychosis history in HD. In addition, younger age at clinical HD diagnosis, lower number of CAG repeats, worse functional capacity and poorer cognitive performance were significant associated with psychosis in HD. An external validation with an independent cohort of patients with HD (Wave 2) and the machine learning approach corroborated these results.

We found that almost 11% of patients from the Enroll-HD database had a history of psychosis. The prevalence of psychotic symptoms in HD patients varies between 3 and 11% (6). The great variability in psychosis prevalence among different studies probably results from participants' selection criteria. For instance, the selection of an outpatient population reduces the likelihood of observing behavioral symptoms severe enough to require hospitalization and therefore may underestimate the prevalence of psychosis (31). Indeed, institutionalized patients with HD are more likely to have delusions and auditory hallucinations than outpatients (32) as the psychotic symptoms are often the cause for institutionalization

The percentage of patients with HD that have psychotic symptoms may vary depending on the disease stage. It has been reported that delusions and hallucinations are more prevalent in the middle stages of the disease (33, 34). However, our analyses revealed that among HD patients with a history of psychosis, psychotic symptoms preceded the clinical diagnosis of HD in the majority of patients (55.3%). A few studies have pointed out that psychotic symptoms in HD may occur before the clinical diagnosis (35, 36). Early psychotic symptoms and inappropriate behaviors have been described in juvenile HD (37, 38). Supporting this latter observation, our results showed that younger age at clinical HD diagnosis were associated with history of psychosis. Only 18 out of the 2,303 patients from the Enroll-HD dataset (PDS2) had juvenile HD (defined as a clinical diagnosis before 20 years old). From these, 4 had history of psychosis, resulting in a percentage (29%) way above the general HD population. A recent study analyzed data from 230 Spanish patients from the REGISTRY database and found a prevalence of 4% of psychosis in both premanifest and early symptomatic patients with HD (39). Older studies have reported a higher prevalence of schizophrenia-like psychosis in HD in comparison with the general population. Some case-reports described patients that were first diagnosed with schizophrenia-like psychosis and only later with HD (8, 40–42). Taken together, these data confirm that psychosis may antecede motor symptoms in HD.

There are a few studies evaluating clinical correlates of psychosis in HD. A study involving a large cohort of HD mutation carriers (1,993 participants from the observational REGISTRY study) investigating current psychosis, found low percentages of participants scoring mild (2.9%) and moderate to severe (1.2%) psychosis. HD mutation carriers with psychosis had a significantly longer duration of disease, a higher TMS, a lower TFC score, more often a positive psychiatric history for depression, obsessive compulsive behaviors and psychosis, and more often used benzodiazepines and antipsychotics. The only independent correlates of current psychosis were a history of psychosis and use of benzodiazepines (34). Overall these results corroborate our findings.

We also found that lower number of CAG repeats were associated with history of psychosis. The age of clinical onset of symptoms in HD is strongly influenced by the length of the CAG trinucleotide expansion within the *HTT* gene (1). It is possible that since the clinical onset of HD is defined mainly by motor symptoms, the patients with lower CAG repeats may present with psychiatric problems, including psychosis, before the development of significant motor symptoms necessary for clinical diagnosis.

There are specific HD phenotypes that represent the heterogeneity in clinical presentation and rates of progression (1). The pathophysiological process underlying HD may contribute to the development of psychotic symptoms in a subset of patients. However, because not all HD patients or families are susceptible to developing psychosis, other predisposing factors may also exist (43). Herein, we described clinical factors associated with psychosis in HD, such as psychiatric and behavioral problems and poorer cognitive performance. These factors might predispose psychosis in HD. However, genetic/biological factors might also contribute to the development of psychosis in HD. Future studies are needed in this regard. The hypothesis that the presence of psychotic symptoms might be part of a unique HD phenotype is substantiated by some studies with HD families. In some HD pedigrees, psychotic symptoms have defined the disease phenotype across generations. In these families, psychosis was the most prominent symptom and preceded the motor or cognitive changes in almost all affected members (8). Another study found that probands with psychotic symptoms were much more likely to have a first-degree relative with psychosis than were the nonpsychotic comparison probands. In addition, the age at onset of psychosis was lower in probands with a higher number of CAG repeats (43).

We are aware of the limitations of our study. First, we cannot make assumptions about the temporal relationship between the factors associated with psychosis and psychosis itself. A longitudinal study is needed in this regard. Also, some variables we described as significant associated with psychosis are difficult to be defined (e.g., drugs of abuse), but this is a limitation intrinsic to the information available in the Enroll-HD dataset. Lastly, our analyses were not controlled for medications use and some drugs might mask psychotic symptoms [e.g., antipsychotics that are commonly used for the management of chorea in HD (44)]. The majority of the patients (*N* = 1,272, 55%) have history of antipsychotics use. A great percentage (*N* = 830, 36%) was using antipsychotics at the time of the baseline interview (the source of information for the current study). The most commonly used antipsychotics were olanzapine (*N* = 228), followed by tiapride (*N* = 160), risperidone (*N* = 128), quetiapine (*N* = 104) and haloperidol (*N* = 69). On the other hand, the big sample size, the use of both conventional statistics and machine learning methods with convergent results and the external validation with an independent cohort of patients with HD can be regarded as strengths of our study. In addition, the multivariate machine learning approach is able to consider interactions between all variables and to distinguish between HD patients with and without psychosis with significant accuracy (specificity and sensitivity). Noteworthy, our results were validated/replicated in an independent cohort (i.e., wave 2 analyses).

Psychiatric and behavioral problems and poorer cognitive performance were significant associated psychosis in HD. Accordingly, psychosis seems to occur in patients with HD that also have a burden of other non-motor symptoms. Further analysis in the larger database with longitudinal assessments will allow refining the analysis and determining predictors of psychosis in HD. As psychosis was associated with lower number of CAG repeats and younger age at clinical diagnosis of HD, our study also suggests that patients presenting with a history of psychosis may represent a unique phenotype in the HD spectrum. A well-defined subtype of HD patients may allow the identification of genetic modifiers associated with newer pathogenic mechanisms and lead to novel therapeutic targets.

## Ethics statement

The study protocol has been approved by the UTHealth Committee for the Protection of Human Subjects (IRB number: HSC-MS-15-0881). A waiver of consent was granted since we worked with a consolidated data set, containing only de-identified data.

## Author contributions

EF and AT worked on the design and conceptualization of the study. NR, BM, and CG worked on the data analysis and interpretation. NR and BM wrote the first draft of the manuscript. EF, CS, and AT revised the manuscript for intellectual content. All authors read and approved the manuscript.

### Conflict of interest statement

The authors declare that the research was conducted in the absence of any commercial or financial relationships that could be construed as a potential conflict of interest.

## Supplementary material

Supplementary Figure 1 Receiver Operating Characteristic (ROC) curve for the binary logistic regression. **(A)** The goodness of fit of the logistic regression model was assessed by a ROC curve, which resulted in an area under the curve (AUC) of 0.793 for the Wave 1 analysis. **(B)** The ROC curve for the binary logistic regression with an independent cohort of patients with HD (external validation, Wave 2 analysis) resulted in an AUC of 0.816.

Supplementary Table 1 Validation analysis: final logistic regression model (step 18) to define factors associated with psychosis in Huntington's disease (HD) in the Wave 2 sample (PDS3; i.e., new data released at the Enroll-HD periodic dataset containing information as of October 31, 2016, *N* = 2,564).

Supplementary Table 2 Scores obtained by patients with HD with and without history of psychosis in Problem Behaviors Assessment-short version (PBA-s).
