# Supplementary figures and images for: The Clinical Picture of Psychosis in Manifest Huntington's Disease: A Comprehensive Analysis of the Enroll-HD Database
Source: Front Neurol. 2018 Nov 6;9:930. doi: 10.3389/fneur.2018.00930 (PMC6232301; doi:10.3389/fneur.2018.00930)

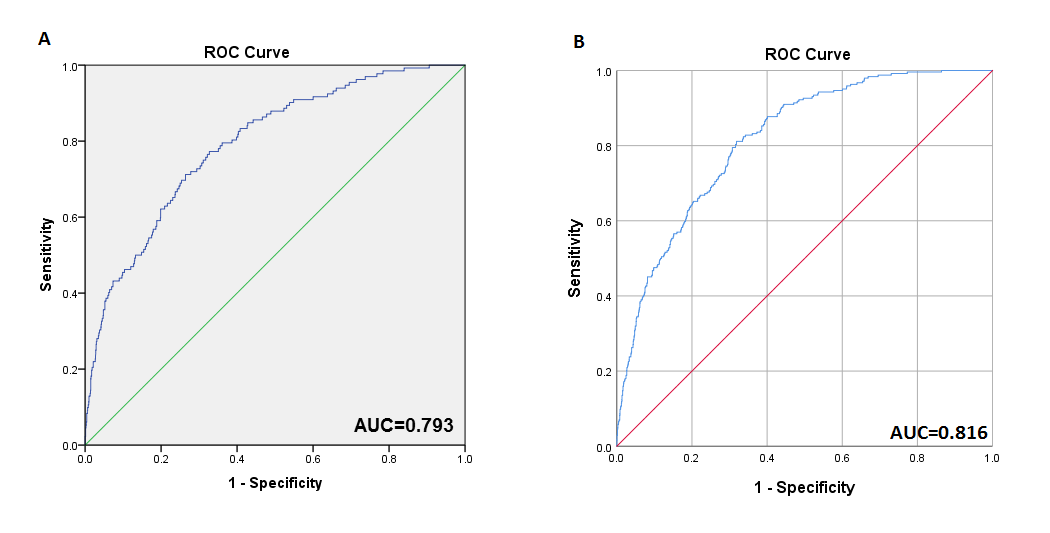

Supplement: Supplementary Figure 1 — Receiver Operating Characteristic (ROC) curve for the binary logistic regression. (A) The goodness of fit of the logistic regression model was assessed by a ROC curve, which resulted in an area under the curve (AUC) of 0.793 for the Wave 1 analysis. (B) The ROC curve for the binary logistic regression with an independent cohort of patients with HD (external validation, Wave 2 analysis) resulted in an AUC of 0.816. [file Image_1.TIF]
