# Supplementary material for: The Clinical Picture of Psychosis in Manifest Huntington's Disease: A Comprehensive Analysis of the Enroll-HD Database
Source: Front Neurol. 2018 Nov 6;9:930. doi: 10.3389/fneur.2018.00930 (PMC6232301; doi:10.3389/fneur.2018.00930)
Supplement: Supplementary Table 1 — Validation analysis: final logistic regression model (step 18) to define factors associated with psychosis in Huntington's disease (HD) in the Wave 2 sample (PDS3; i.e., new data released at the Enroll-HD periodic dataset containing information as of October 31, 2016, N = 2,564). [file Table_1.DOCX]

**Supplementary tables**

**Supplementary Table 1.** Validation analysis: **f**inal logistic regression model (step 18) to define factors associated with psychosis in Huntington’s disease (HD) in the Wave 2 sample (PDS3; i.e., new data released at the Enroll-HD periodic dataset containing information as of October 31, 2016, N = 2,564).

| **Variable** |  |  |  |  |  |  | **95% CI for Odds Ratio** | |
| --- | --- | --- | --- | --- | --- | --- | --- | --- |
|  | **B** | **SE** | **Wald** | **df** | **p Value** | **Odds Ratio** | **Lower** | **Upper** |
| Age at clinical diagnosis | -0.044 | 0.017 | 6.645 | 1 | 0.010 | 0.957 | 0.926 | 0.990 |
| Number of CAG repeats | -0.179 | 0.059 | 9.107 | 1 | 0.003 | 0.836 | 0.744 | 0.939 |
| History of suicidal ideation | 0.653 | 0.246 | 7.043 | 1 | 0.008 | 1.921 | 1.186 | 3.112 |
| History of alcohol use disorders | 0.839 | 0.309 | 7.378 | 1 | 0.007 | 2.314 | 1.263 | 4.240 |
| History of violent/aggressive behavior | 0.755 | 0.249 | 9.163 | 1 | 0.002 | 2.127 | 1.305 | 3.468 |
| History of apathy | 0.508 | 0.294 | 9.163 | 1 | 0.084 | 1.662 | 0.933 | 2.958 |
| History of perseverative/obsessive behavior | 0.818 | 0.255 | 10.321 | 1 | 0.001 | 2.266 | 1.376 | 3.734 |
| SDMT (number of correct responses) | -0.025 | 0.013 | 3.579 | 1 | 0.059 | 0.975 | 0.950 | 1.001 |
| TFC score | -0.167 | 0.049 | 11.503 | 1 | 0.001 | 0.846 | 0.769 | 0.932 |

Abbreviations: CI = confidence interval; df = degrees of freedom; TFC = total functional capacity; SDMT = symbol digit modalities test; SE = standard error.

**Supplementary Table 2.** Scores obtained by patients with HD with and without history of psychosis in Problem Behaviors Assessment – short version (PBA-s).

|  | **Psychosis history** | | *P* value |
| --- | --- | --- | --- |
|  | **No (N=2055)** | **Yes (248)** |  |
| Depression | 4.78 ± 6.15 (2) | 7.14 ± 7.37 (5.5) | **0.000^a^** |
| Irritability/aggression | 3.24 ± 4.87 (1) | 5.22 ± 6.66 (3) | **0.000^a^** |
| Psychosis | 0.10 ± 0.72 (0) | 2.42 ± 5.21 (0) | **0.000^a^** |
| Apathy | 3.49 ± 4.50 (1) | 6.68 ± 5.54 (6) | **0.000^a^** |
| Executive function | 3.25 ± 5.25 (0) | 8.82 ± 9.00 (6) | **0.000^a^** |

^a^Mann-Whitney test.
